# Dynamic Changes and Influencing Factors of Vegetation in the “Green Heart” Zone of the Chang-Zhu-Tan Urban Agglomeration during the Past 21 Years

**DOI:** 10.3390/ijerph20054517

**Published:** 2023-03-03

**Authors:** Chaokui Li, Rui Zhang, Ting Li, Haibin Guo, Ruirong Guo

**Affiliations:** 1 Hunan Provincial Key Laboratory of Information Engineering for Surveying, Hunan University of Science and Technology, Xiangtan 411201, China; 2 National and Local Joint Engineering Laboratory of Geospatial Information Technology, Hunan University of Science and Technology, Xiangtan 411201, China; 3 College of Earth Science and Spatial Information Engineering, Hunan University of Science and Technology, Xiangtan 411201, China; 4 College of Architecture and Artistic Design, Hunan University of Science and Technology, Xiangtan 411201, China

**Keywords:** green heart area, NDVI, temporal and spatial changes, geographical detectors, influence factor

## Abstract

As a policy, protected green space in the rapidly developing the Chang-Zhu-Tan Urban Agglomeration is of great practical significance to study the vegetation changes and influencing factors in the Green Heart area. In this paper, data processing, grading and area statistics were carried out for the maximum value of normalized differential vegetation index (NDVI) from 2000 to 2020. Combined with Theil–Sen median trend analysis and Mann–Kendall, the change trend of long-time series NDVI was studied, and investigation of NDVI influencing factors, processes and mechanisms using geographical detectors. The results showed that: (1) The spatial distribution characteristics of NDVI in the study area were high in the middle and inlaid transition between adjacent grades. Except for the low grades, the distribution of NDVI in other grades was relatively scattered, and the overall trend of NDVI change was rising. (2) Population density was the main factor affecting NDVI changes, with an explanatory power of up to 40%, followed by elevation, precipitation and minimum temperature. (3) The influence of influencing factors on the change of NDVI was not the result of independent action of a single factor, but the result of the interaction between human factors and natural factors, and the factor combinations with greater interaction had significant differences in the spatial distribution of NDVI.

## 1. Introduction

Vegetation is an important part of the Earth’s ecosystem, an important carrier of photosynthesis, and provides energy for human production and life [1,2,3,4]. Among some parameters used to evaluate the growth state of vegetation, NDVI is used most frequently, and is often used in the research of carbon emission change, land use type research, environmental monitoring, vegetation change monitoring, etc. [5,6,7,8]. The value of NDVI is always between −1 and 1. When the value is closer to 1, it indicates that the vegetation density is higher and, on the contrary, the vegetation coverage is lower [9,10,11,12]. Due to different precipitation, temperature and sunshine duration in different months, the growth of vegetation in a year is different. Taking the maximum value of NDVI in a year can effectively reflect the best state of vegetation growth. This method is widely used in the study of vegetation change [13,14,15,16].

In recent years, many scholars have studied vegetation cover change and its influencing factors through different methods. Some scholars have mainly studied the relationship between precipitation, soil moisture and the maximum NDVI in arid and semi-arid areas, using correlation analysis, and the results showed that there is no significant simple proportional relationship between the maximum NDVI and the two meteorological data [17,18,19,20,21]. Some researchers used the comprehensive analysis model of multi-component vegetation directional reflection coefficient to deeply study the influence of vegetation structure and sun angle on NDVI [22,23,24,25,26,27]; some scholars analyzed the influencing factors of NDVI in different regions from two aspects of natural and anthropic factors through trend surface analysis and correlation analysis, and concluded that NDVI was affected by human activities [28,29,30]; some researchers carried out a multi time scale analysis on NDVI of long-time series, mainly analyzing the spatio-temporal variation trend of NDVI and the rule of plant growth cycle [31,32,33,34]. Benewinde selected accessibility factors, climate factors and terrain factors to analyze the influencing factors of West African vegetation and concluded that most of the negative changes in natural vegetation occurred at the boundary of the reserve and in the fragmented landscape characterized by the continuous destruction of natural vegetation, and the three factors led to the degradation of vegetation [35].

The Chang-Zhu-Tan Urban Agglomeration is an important part of the urban agglomeration in the middle reaches of the Yangtze River, it is the fourth batch of metropolitan areas approved by the National Development and Reform Commission for development. The Green Heart area is located at the junction of Changsha, Zhuzhou and Xiangtan. With an important geographical location, it is the key ecological protection area of the Chang-Zhu-Tan Urban Agglomeration. At present, some studies have analyzed the vegetation coverage and influencing factors of the Chang-Zhu-Tan Urban Agglomeration [36,37,38]. For example, Li analyzed the vegetation coverage in some areas of Changsha Zhuzhou Xiangtan in 2000 and 2016, and concluded that the vegetation was greatly affected by humanistic factors [39]. From the perspective of urban expansion, we analyzed its impact on vegetation change. Using trend surface analysis, it was concluded that from 2001 to 2017, the vegetation index in the surrounding areas of Chang Zhu Tan decreased slowly, and the Green Heart area was better protected in the process of urban expansion [40].

Since the Green Heart District put forward the ecological construction and development goal of “overall improvement in the quality of the ecological environment” in 2012, the district has vigorously built ecological infrastructure, and actively implemented projects such as returning farmland to forests and grass, and planting trees and grass on slopes. However, what is the impact of the implementation of ecological engineering on the vegetation development in the Green Heart area? Has the implementation of the project accelerated the rate of ecological restoration in the Green Heart area? Is the restoration effect and speed of the ecological environment quality in the Green Heart District better than that of the surrounding urban areas? The answers to these questions are unknown. Therefore, it is of far-reaching significance for urban management and planning strategies to conduct long-term monitoring and evaluation studies on the vegetation in the Green Heart area, monitor and quantify the temporal and spatial evolution characteristics of the ecological environment in the Green Heart area, and explore its driving factors. In order to solve the above problems, this study introduces geographic detectors to design a set of widely applicable new evaluation methods for the study of spatial-temporal changes in green-heart vegetation. This study analyzes the temporal and spatial changes of NDVI in the study area in the past twenty-one years (2000–2020) and selects fourteen factors from two aspects of nature and humanities, and uses geographic detectors to analyze the impact mechanism of NDVI in the Green Heart area. The research results have important guiding significance for the planning and construction of the Green Heart area and the monitoring of the ecological environment, and provide a scientific decision-making basis for the sustainable development and protection of the ecological environment in the area. It also provides a new idea for the evaluation of ecological environment quality in other regions.

## 2. Materials and Methods

### 2.1. Study Area

**Figure 1:**
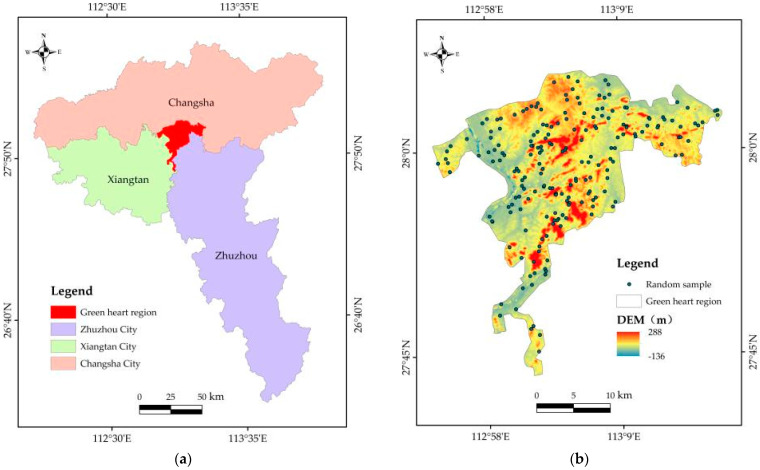
(**a**) Location of Green Heart in the Chang-Zhu-Tan Urban Agglomeration; (**b**) Elevation diagram of green heart.

The Green Heart area is located at 112°53′ E~113°18′ E, 27°43′ N~28°7′ N (Figure 1), with a total area of 528.32 km^2^. The Green Heart area is located at the central junction of Changsha, Zhuzhou and Xiangtan. Changsha Ring Road and Liuyang River are in the northernmost part of the Green Heart area, reaching Yisuhe town of Xiangtan County in the south, Liuyang town in the East, and the farthest west line of the Changsha–Xiangtan Expressway in the West. The areas of Changsha, Zhuzhou and Xiangtan are 306.00 km^2^, 83.87 km^2^ and 138.45 km^2^, respectively. The elevation of the Green Heart area is between −136~288 m, the terrain is high in the middle and low around, there is abundant precipitation, and water resources are abundant. The annual maximum temperature is 36~41 °C, the annual average temperature is 16~19 °C, the annual minimum temperature is −6~0 °C and the annual total sunshine hours are 1212~1616 h. There are mainly some sweet-scented osmanthus trees and plane trees here, and mainly evergreen broad-leaved forests. In recent years, under the protection of policies, the ecological environmental quality of Green Heart areas has gradually improved.

The NDVI data used in this research from 2000 to 2020 were from the National Science and Technology Resource Sharing Service Platform (http://www.nesdc.org.cn/ (accessed on 5 June 2022)), this data set obtained the maximum value of NDVI through linear interpolation and S-G smoothing. The value of NDVI was between 0–255, the spatial resolution was 30 m, and the temporal resolution was year [41]. After normalization, the value of NDVI was between −0.2–1.

The data of land use, precipitation, temperature, humidity, sunshine, wind speed and pressure were from the National Earth System Science Data Center (http://www.geodata.cn/ (accessed on 5 June 2022)), population density data were from worldpop (https://www.worldpop.org/ (accessed on 5 June 2022)). The night light data came from Harvard’s dataverse platform (https://dataverse.harvard.edu/ (accessed on 5 June 2022)). DEM data came from geospatial data cloud (http://www.gscloud.cn/ (accessed on 5 June 2022)), slope and aspect data were generated from DEM.

### 2.2. Research Methods

#### 2.2.1. Theil–Sen Median Trend Analysis

Theil–Sen median trend analysis is a nonparametric statistical method that is often used to analyze geographical phenomena with long-time series changes [42,43,44]. Its advantage is that it is less affected by abnormal factors [45,46]. This study used this statistical method to analyze the continuous change trend of NDVI from 2000 to 2020. In the following formula, *y_a_* and *y_b_* represent the maximum NDVI in years *a* and *b*, respectively. When it is greater than zero, it indicates an upward trend; when it is less than zero, it indicates a downward trend.

(1) $$\beta =median\left(\frac{y_{a}-y_{b}}{a-b}\right),\;1<b<a<n$$

#### 2.2.2. Mann–Kendall

The MK test method is usually used to detect the sudden changes in climate and determine the sudden change time, which is suitable for testing of long-time series changes [47,48,49,50,51]. In this paper, the trend results of Theil–Sen median were graded by MK test method. *S* is the statistic, *Z* is the test statistic, the NDVI values of years *i* and *j* years are expressed by *x_i_* and *x_j_*, respectively, and the number of data is expressed by *n*.

(2) $$S=\overset{n-1}{\underset{i=1}{\sum }}\overset{n}{\underset{j=i+1}{\sum }}sgn\left(x_{j}-x_{i}\right)\;\;\;\;\;\;\;sgn\left(x_{j}-x_{i}\right)=\left\{\begin{array}{c} +1(x_{j}-x_{i}>0)\\ 0\left(x_{j}-x_{i}=0\right)\\ -1(x_{j}-x_{i}<0) \end{array}\right.$$

(3) $$Z=\frac{s}{\sqrt{Var\left(s\right)}}(s>0)\;Z=0\left(s=0\right)\;Z=\frac{s+1}{\sqrt{Var\left(s\right)}}(s<0)\;\;Var\left(s\right)=\frac{n\left(n-1\right)\left(2n+5\right)}{18}$$

Combining the above two methods, the results are divided into 9 grades. When *β* > 0, and *z* > 2.58, it is extremely significantly increased, 1.96 < *z* ≤ 2.58 is significantly increased, 1.65 < *z* ≤ 1.96 is slightly significantly increased, and *Z* ≤ 1.65 is not significantly increased. When *β* = 0, *z* = 0, there is no change; *β* < 0 is the opposite of *β* > 0. Classification is shown in Table 1

#### 2.2.3. Geographic Detectors

Geographic detectors are used to analyze spatial differentiation [52,53], and consist of four parts. This paper used three parts, factor detection, interactive detection and ecological detection, to study the influencing factors of NDVI from 2000 to 2020. Compared with traditional analysis methods, the advantage of geographical detector is that it can analyze the influence of multiple factors on the same object. Factor detection uses *q* value to express the influence of influencing factors on NDVI. The greater the *q* value indicates the greater influence. Interactive detection evaluates the influence of multiple influencing factors on NDVI. Ecological detection evaluates whether there are significant differences between multiple influencing factors on the spatial distribution of NDVI. *L* is the total number of categories of factors, *h* is a certain type of factor, *Nh* means: *N* is the total number of units; it is the variance of class *h* (total variance). According to the value of NDVI, it is divided into five levels: low (≤0.2), lower (0.2–0.4), medium (0.4–0.6), higher (0.6–0.8), high (≥0.8).

(4) $$q=1-\frac{\sum_{h=1}^{L}N_{h}\sigma_{h}^{2}}{N\sigma^{2}}$$

When selecting the influencing factors, many factors were considered. First, the study area has rich types of vegetation and complex and diverse natural factors. Therefore, vegetation is inseparable from various natural factors. However, vegetation and various natural factors are not in a closed static relationship. The two are connected and interact with each other in a certain time and space, and there is an inseparable exchange of matter, energy and information between them. The coordinated development between vegetation and natural factors will promote the improvement of vegetation structure and scale, and provide a good foundation for the ecological environment, otherwise it will hinder the cyclic development of the entire ecological environment.

Secondly, although the evolution of vegetation is mainly affected by natural factors in the long run, in the short run, vegetation is greatly disturbed by human activities. Vegetation, especially forestry, is cut down as a natural resource, and a large amount of wood is consumed for household and industrial fuels, and wood is used to process and manufacture a large number of industrialized industries, which promotes regional economic development. However, excessive deforestation will cause the mountains to no longer be covered by forests, causing serious ecological and environmental problems such as water quality decline, soil erosion, biodiversity loss and climate change. Ultimately, this chain of effects forms a vicious circle among urbanization, population growth, economic development and deforestation.

Vegetation is inseparable from natural factors and human factors. First, vegetation is an important basis for economic and social activities. Vegetation provides a variety of resources for human economic and social activities, and people use the materials provided by vegetation to produce and live. On the one hand, people rely on vegetation to obtain raw materials and energy, on the other hand, people use them and return them to the ecosystem in the form of waste, and directly or indirectly affect the growth of vegetation. Second, economic and social activities affect vegetation and determine the development direction of vegetation coverage. Human behavior can have a strong impact on vegetation and how it evolves. Humans use vegetation resources mainly to meet human needs. If this need exceeds a certain limit, the vegetation state will transition from a stable state to an unstable state. At this time, vegetation resources may not be able to provide services for humans and may even limit human economic and social activities. In addition, human beings can also improve the level of vegetation coverage through modern institutional measures, so as to coordinate the development between economic and social development and the ecological environment. In summary, 14 influencing factors were selected, as shown in Table 2.

## 3. Results

### 3.1. Spatial and Temporal Changes of NDVI

#### 3.1.1. Spatial and Temporal Variation Characteristics of NDVI

**Figure 2:**
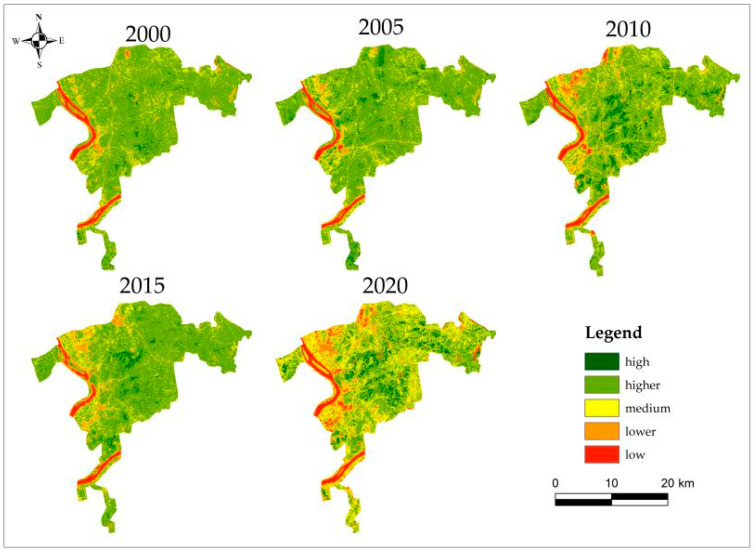
NDVI distribution map of each year.

The NDVIs of 2000, 2005, 2010, 2015 and 2020 were selected to analyze the change characteristics from the time scale. The results presented in Table 3 showed that the area of low and high grade NDVI in each year accounted for a relatively small proportion, followed by the area of lower grade NDVI, and the area of medium and higher grade NDVI accounted for a relatively large proportion. From 2000 to 2020, the area of low grade NDVI fluctuated and increased, with the largest area of 36.92 km^2^ in 2020. The area of lower grade NDVI continued to increase, and the growth rate was the largest in the five years from 2015 to 2020. The area of medium grade NDVI exceeded 100 km^2^ in 2010 and 2020. The area of higher grade NDVI was more than 300 km^2^ except in 2020, which showed a decrease in volatility (area fluctuation). The high grade NDVI reached the highest level in 2020, between 10 km^2^ and 35 km^2^. It can be seen from Figure 2 that from 2000 to 2020, NDVI with high vegetation density was distributed in the middle and gradually spread eastward. The higher grades were widely distributed and evenly distributed in the study area, which decreased more in 2020, and most of the reduced parts became medium grades. The middle grade part was gathered around the river and distributed in a mosaic with the lower grade. The low grade was mainly distributed in the west and south, gathered in the river, in a sinuous shape.

#### 3.1.2. Spatial and Temporal Variation Trend of NDVI

**Figure 3:**
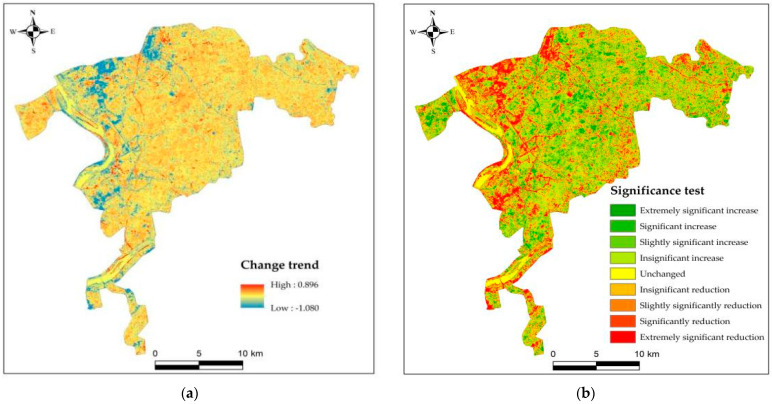
(**a**) NDVI change trend from 2000 to 2020; (**b**) NDVI significance test from 2000 to 2020.

Figure 3 shows that in the continuous NDVI change trend from 2000 to 2020, the upwards trend accounted for 53.46% of the total area, which was scattered in the study area. Among them, the extremely significant increase accounted for 6.83% of the total area, the significant increase accounted for 9.04%, the slightly significant increase accounted for 5.98% and the nonsignificant increase accounted for 31.62%. NDVI without trend change was mainly distributed in and near rivers, accounting for 7.05% of the total area; the downwards trend accounted for 39.49% of the total area, most of which were concentrated in the west of the middle and close to the river. On the whole, there were more areas with no significant increase and no significant decrease, and the part with an upward trend of NDVI was more than the part with a downward trend.

### 3.2. Geographical Detection of NDVI Influencing Factors

#### 3.2.1. Factor Detection of Influencing Factors

According to the factor detection results in 2020, the *q* value was sorted from large to small: population density (0.43) > elevation (0.30) > precipitation (0.25) > minimum temperature (0.24) > maximum temperature (0.22) > average temperature (0.19) > slope (0.18) > wind speed (0.16) > sunshine hours (0.12) > night light (0.11) > air pressure (0.06) > relative humidity (0.04) > slope direction (0.02) > land use (0.01). The *q* value of population density and elevation was greater than or equal to 0.3. The *q* value of precipitation, minimum temperature, maximum temperature, average temperature, slope, wind speed, sunshine hours and night light was between 0.1–0.3, which was the secondary factor affecting the change of NDVI. The *q* value of relative humidity, slope aspect, air pressure and land use was less than 0.1, which had a weak impact on NDVI changes.

**Figure 4:**
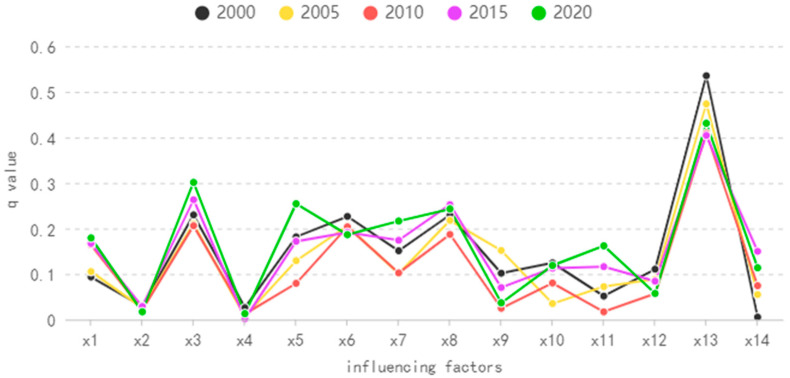
*Q* value results of each year.

According to the factor test results in 2000, 2005, 2010, 2015 and 2020 (Figure 4), the population density among human factors had the greatest impact on the NDVI in 2000. By 2020, the impact intensity weakened, but the explanatory power was still more than 40%. The impact intensity of night light on NDVI gradually increased, and the *q* value in 2015 and 2020 exceeded 0.10. The influence of topographic factors, elevation and slope on NDVI continued to become stronger, reaching the maximum in 2020, and the influence of slope direction on NDVI was small and changed little. In terms of climatic factors, the influence degree of precipitation on NDVI was the lowest in 2010 and the highest in 2020, which was generally in a strengthened state. The influence degree of the minimum temperature and the maximum temperature on the NDVI had a fluctuating increasing trend. The *q* value of the average temperature gradually decreased, but the *q* value was above 0.19. The influence of wind speed and air pressure on NDVI was in a fluctuating stronger state, and the *q* value of sunshine hours first decreased and then increased, which was generally unchanged; the variation in the *q* value of air pressure and relative humidity decreased in fluctuation.

#### 3.2.2. Interactive Detection of Influencing Factors

Interaction detection is mainly used to analyze the results of the joint action of two factors on NDVI. For the fourteen factors selected in this study, there were two interaction results. One was double factor enhancement, i.e., the influence of the two factors on NDVI is greater than the maximum value of the two factors acting alone; and the other was nonlinear enhancement, i.e., the influence of the two factors on NDVI is greater than the sum of the influence of the two factors alone.

It can be seen from Table 4 that the value of population density and slope was the largest when they acted together, which was 0.62, and the explanatory power was up to more than 60%, which belonged to double factor enhancement. The interaction value of the bold part in Table 4 was greater than 0.5. Second, x1 ∩ x3, x10 ∩ x11, x4 ∩ x13, the above interaction values were between 0.45–0.50. Last, the interaction values of the italic parts in Table 4 were greater than 0.4 and less than 0.45.

#### 3.2.3. Ecological Detection of Influencing Factors

In Table 5, Y represents that there was a significant difference in the spatial distribution of NDVI between factor combinations, while N represents that there was no significant difference. The spatial distribution of NDVI in human factors and other 13 factors was Y, and there were significant differences in the spatial distribution of NDVI in night light and elevation. Among the natural factors, the results of slope aspect and elevation, precipitation and three temperature factors in terrain were expressed as Y. There were significant differences between elevation and land use, relative humidity, sunshine hours and air pressure on the spatial distribution of NDVI. In terms of climate, the ecological detection results of precipitation and relative humidity and pressure, temperature and relative humidity and pressure were also Y; land use, precipitation and temperature had significant differences in the spatial distribution of NDVI, and the results between other factors were expressed as N, indicating that there was no significant difference in the spatial distribution of NDVI.

## 4. Discussion

This study extracted the maximum NDVI of each year from 2000 to 2020, and analyzed the vegetation coverage of the long-time series in the Green Heart area. With the development of society, the vegetation coverage in the areas where human activities were concentrated gradually decreased, and the areas of low and lower NDVI gradually increased. In 2015, the areas of low and lower NDVI showed a decrease in volatility. The medium grade showed increased volatility, mainly distributed in flat terrain. NDVI areas of high and higher grades were mainly distributed in areas with higher elevations. On the whole, the area of NDVI with an upward trend was 13.97% more than that with a downward trend, and the area of NDVI with a very significant increase and a significant increase was more than that with a very significant decrease and a significant decrease; the change of vegetation showed a good trend. In 2013, Hunan Province issued a protection policy for the Green Heart area of the Chang-Zhu-Tan Urban Agglomeration, which is divided into three zones, namely the prohibited development zone, the restricted development zone and the controlled construction zone. This policy greatly restricts the expansion of human construction in the Green Heart area and strictly controls the construction of the Green Heart area. Due to the reduction in construction activities, the vegetation coverage was reduced slowly, and the ecosystem was restored, and the change of vegetation cover also showed an upward trend.

At the same time, it was observed from the spatial distribution of NDVI changes that the significant reduction in NDVI was concentrated in the upper left corner of the study area, which is close to Changsha City, and the city’s outward expansion is the main reason for its changes. The development of the central urban area requires a large amount of land in and around the city, which has led to the transformation of a large number of vegetation land types such as cultivated land, grassland and woodland into impermeable layers, destroying the ecological environment. For cities, better planning and development of construction capabilities are crucial in order to improve the urban environment, mitigate negative effects and adapt to climate change. According to the theory of sustainable development, if governments at all levels want to achieve the goal of urban sustainable development, they need to improve the institutional capacity of urban planning and management.

From the perspective of a single factor, it is mainly affected by population density, which represents the intensity and frequency of human activities. The higher the population density, the smaller the value of NDVI, and human activities lead to the reduction in vegetation. The second factor is elevation, precipitation and minimum temperature. The higher the elevation influenced, the greater the NDVI value. The precipitation and minimum temperature in the study area were in direct proportion to the NDVI value. According to previous studies, precipitation and temperature can provide the necessary conditions for vegetation growth and promote the growth of vegetation. From the perspective of time series, because policies restrict human mining activities, the impact of population density in human factors on NDVI changes is weakened, and the impact of elevation, slope, precipitation and temperature in natural factors on NDVI changes is enhanced. From the perspective of two factors, population density and slope have had the greatest impact on the change of NDVI, and there are significant differences in the spatial distribution of NDVI. From the pairwise interaction results of 14 factors, there were following three cases: first, the factors acted independently on the change of NDVI; second, the interaction was less than the minimum value of two factors acting alone; and third, the value of interaction was between the values of two factors acting alone. It shows that the influencing factors of NDVI change were the result of the joint action of human factors and natural factors.

The new research approach proposed in this study on vegetation change in the green heart will help to establish a living environment in the Green Heart area of the urban agglomeration that prioritizes the ecological function of the land and protects the ecological benefits of the land. At the same time, it conforms to the current development theme of ecological economy and green economy and promotes the construction of a resource-saving and environmentally friendly society in the Green Heart area. At the same time, it has a significant effect on the protection and utilization of ecological resources in the green center of Changsha-Zhuzhou-Tan, and the promotion of ecological production, social coordination and sustainable development of the Changsha-Zhuzhou-Tan Urban Agglomeration. As the ecological green space of the Chang-Zhu-Tan Urban Agglomeration, it is necessary to comprehensively and deeply understand the vegetation coverage and the driving mechanism of influencing factors in the Green Heart area. The planning and construction of the Changsha Zhuzhou Xiangtan Lvxin area is still in progress; the research results are expected to become the reference basis in the governance and planning process of the Chang-Zhu-Tan Green Heart area, and provide more targeted and directional protection for the ecology of the Green Heart area.

At present, due to the unprecedented scale and speed of integration of the Changsha-Zhuzhou-Tan Urban Agglomeration, the impact on the ecological system of the Green Heart area, especially the destruction of vegetation, is not only reflected in the edge of the area, but also seriously damages a wide range of forests. These ecological and environmental problems, including vegetation, are complex and diverse, involving various spatial scales from cities to communities. It is impossible to achieve sustainable goals if each area is only managed individually. Since the nodes in the middle of urban agglomerations are usually separated by rural areas, and small-scale urban areas generally lack green facilities and funds, and a single area can only improve its own environment. However, vegetation destruction often causes cross-regional problems such as soil erosion, climate change and biodiversity, which require a wider range of governance forms, management mechanisms and the adaptation of more people’s technology and funds. Evidently, the improvement of vegetation requires the close integration of vegetation improvement policies, standards and practices in various regions. It is necessary to greatly strengthen regional cooperation, coordination and commitment to jointly promote the ecological protection and sustainable development of the green heart of urban agglomerations.

## 5. Conclusions

This paper conducted an in-depth study on the spatial-temporal change characteristics of vegetation coverage in the Changzhou-Zhuzhou-Tan metropolitan area and the quantitative analysis of the driving factors in the past 21 years. The correlation and significance between the main influencing factors in the region and NDVI were calculated by using geographic detectors to calculate the driving forces of related natural and human factors, and the main driving factors of NDVI changes in the region were obtained. Compared with many existing similar studies, this paper quantitatively calculated the driving forces of a series of factors affecting NDVI changes in the green center area with the help of geographic detectors, and calculated the driving force values under the interaction of two factors. At the same time, this paper quantitatively analyzed the impact of human activities, and obtained the specific driving force of the factors. This study mainly drew the following conclusions:

(1) From 2000 to 2020, NDVI showed an obvious change trend, with an overall upward trend. The vegetation coverage of the river and surrounding areas is stable, belonging to low grade. The distribution characteristics of NDVI are high in the middle, and the distribution between adjacent grades is mosaic. Due to the protection of the policy, the vegetation coverage level in Lvxin area is higher than that in the surrounding areas.
(2) The influence degree of each factor on NDVI is as follows: population density, elevation, precipitation, minimum temperature, maximum temperature, average temperature, slope, wind speed, sunshine hours, night light, air pressure, relative humidity, slope direction and land use. Among them, the main influencing factors are population density in human factors and elevation in natural factors, with an explanatory power of more than 30%.
(3) Among the 91 combinations of factors, 35.16% of the combinations had significant differences in the spatial distribution of NDVI, 14.28% of the combinations had a value of more than 0.50 when interacting, and 21.97% of the combinations had a value of 0.4–0.5 when interacting.

## Figures and Tables

**Table 1:** Gradation.

The Value Range of β	The Value Range of *z*	Category Level
β > 0	*z* > 2.58	extremely significant increase
	1.96 < *z* ≤ 2.58	significant increase
	1.65 < *z* ≤ 1.96	slightly significant increase
	*Z* ≤ 1.65	not significant increase
β = 0	*z* = 0	slightly significant increase
β > 0	*z* > 2.58	extremely significant decline
	1.96 < *z* ≤ 2.58	significant decline
	1.65 < *z* ≤ 1.96	slightly significant decline
	*Z* ≤ 1.65	not significant decline

**Table 2:** Selection of factors.

Type	Factor Name			
Natural	Slope (X1)	Slope direction (X2)	Altitude (X3)	Land use (X4)
	Precipitation (X5)	Average temperature (X6)	Maximum temperature (X7)	Minimum temperature (X8)
	Relative humidity (X9)	Sunshine hours (X10)	Wind speed (X11)	Pressure (X12)
Humanity	Population density (X13)	Night light (X14)		

**Table 3:** NDVI area statistics of each year (Unit: km^2^).

	2000	2005	2010	2015	2020
low	20.74	17.77	25.83	16.33	36.92
lower	17.85	17.86	32.42	31.15	54.71
medium	76.34	80.17	103.15	76.31	181.65
higher	402.47	391.05	335.40	382.36	221.81
high	10.92	21.47	31.52	22.17	33.23
total	528.32	528.32	528.32	528.32	528.32

**Table 4:** Interactive detection results.

	x1	x2	x3	x4	x5	x6	x7	x8	x9	x10	x11	x12	x13	x14
x1	0.18													
x2	0.31	0.02												
x3	0.46	0.37	0.30											
x4	0.22	0.09	0.34	0.01										
x5	0.39	0.51	*0.41*	0.32	0.25									
x6	*0.40*	0.32	*0.41*	0.23	0.34	0.19								
x7	*0.43*	0.32	*0.41*	0.26	0.32	0.26	0.22							
x8	*0.45*	0.40	*0.41*	0.31	0.35	0.26	0.27	0.24						
x9	0.27	0.24	0.36	0.12	*0.42*	0.39	*0.41*	*0.42*	0.04					
x10	0.33	0.33	0.45	0.21	0.35	0.31	0.34	0.36	0.34	0.12				
x11	0.34	*0.43*	0.47	0.22	0.36	0.38	0.38	0.36	0.30	0.26	0.16			
x12	0.30	0.21	*0.44*	0.13	0.33	0.30	0.35	*0.41*	0.23	0.28	0.36	0.06		
x13	0.62	**0.53**	**0.59**	0.45	**0.54**	**0.52**	**0.53**	**0.53**	**0.51**	**0.54**	0.55	**0.52**	0.43	
x14	0.31	0.30	*0.41*	0.17	0.37	0.39	*0.41*	0.39	0.21	0.27	0.27	0.27	0.52	0.11

The interaction value of the bold part is greater than 0.5, and the interaction value of the italic part is greater than 0.4 and less than 0.45.

**Table 5:** Ecological detection results (95% confidence level).

	x1	x2	x3	x4	x5	x6	x7	x8	x9	x10	x11	x12	x13	x14
x1														
x2	N													
x3	N	Y												
x4	N	N	Y											
x5	N	Y	N	Y										
x6	N	Y	N	Y	N									
x7	N	Y	N	Y	N	N								
x8	N	Y	N	Y	N	N	N							
x9	N	N	Y	N	Y	N	Y	Y						
x10	N	N	Y	N	N	N	N	N	N					
x11	N	N	N	N	N	N	N	N	N	N				
x12	N	N	Y	N	Y	N	N	Y	N	N	N			
x13	Y	Y	Y	Y	Y	Y	Y	Y	Y	Y	Y	Y		
x14	N	N	Y	N	N	N	N	N	N	N	N	N	Y	

## Data Availability

Not applicable.
